# Temporal dynamics of glomerular and microvascular remodeling in high-altitude renal injury: a structure-function analysis in rats

**DOI:** 10.3389/fphys.2026.1801934

**Published:** 2026-06-22

**Authors:** Meng Jia, Quzhen Jimu, Suolang Deji, Yidan Guo, Huaying Wei, Zhihua Shi, Xiaoling Zhou, Ruiji Wang

**Affiliations:** 1 Department of Nephrology, Beijing Shijitan Hospital, Capital Medical University, Beijing, China; 2 Department of Nephrology, Lhasa People’s Hospital, Lhasa, China; 3 Department of Respiratory and Critical Care Medicine, Beijing Shijitan Hospital, Capital Medical University, Beijing, China

**Keywords:** high-altitude, hypobaric hypoxia, KIM-1, microvascular rarefaction, peritubular capillaries, renal adaptation, structure-function uncoupling

## Abstract

**Introduction:**

The temporal dynamics of renal acclimatization during the sub-acute transition to high altitude remain poorly defined. Specifically, the relationship between structural integrity and functional adaptation under sustained hypobaric hypoxia is unclear. This study aims to characterize the time-dependent trajectories of renal remodeling and identify the critical biological window for potential intervention.

**Methods:**

Male Sprague-Dawley (SD) rats were randomized to a normobaric normoxia control group or a simulated hypobaric hypoxia group (5,000 m, PO2: 11.3 kPa) for 3, 7, 14, and 28 days (n=6/group). Renal filtration function was assessed via serum creatinine (CRE) and cystatin C (CysC), while tubular injury and systemic inflammation were evaluated using neutrophil gelatinase-associated lipocalin (NGAL), kidney injury molecule-1 (KIM-1), and interleukin-18 (IL-18). Structural alterations were quantified through hematoxylin and eosin (H&E) and Periodic Acid-Schiff (PAS) staining, with peritubular capillary (PTC) density assessed via immunohistochemistry. The temporal associations and exploratory biomarker discrimination were analyzed using restricted cubic spline (RCS) regression, Spearman correlation, and receiver operating characteristic (ROC) analyses.

**Results:**

Despite stable gross kidney weight and length throughout the exposure, histological analysis revealed progressive microscopic injury. PTC density exhibited a continuous, time-dependent decline that was significantly inversely correlated with tubular injury scores and medullary congestion. Notably, glomerular morphometry exhibited a distinct biphasic response: an initial significant reduction in glomerular diameter at Day 3, followed by subsequent enlargement peaking at Day 14. Temporally, serum biomarkers showed heterogeneous and exploratory trajectories, whereas histological injury remained more persistent. CysC and CRE showed overall temporal variation, but Tukey-adjusted *post hoc* comparisons did not identify significant pairwise differences between individual time points. In the exploratory Day 3 ROC analysis, CRE showed no meaningful early discriminatory ability (AUC = 0.486, 95% CI: 0.124–0.848), whereas KIM-1 showed the highest AUC among the examined circulating biomarkers (AUC = 0.861, 95% CI: 0.641–1.000). However, DeLong pairwise comparisons did not remain statistically significant after Tukey correction, and these ROC findings should be interpreted as hypothesis-generating.

**Conclusion:**

Hypobaric hypoxia induces a distinct state of structure-function uncoupling, characterized by persistent microvascular rarefaction that antedates measurable systemic functional decline. The first week of exposure represents a biologically active phase of vascular remodeling and tubular stress. These findings suggest that relying solely on functional markers may underestimate the severity of sub-clinical renal injury in hypoxic environments, highlighting the potential value of integrating structural biomarkers for more accurate risk stratification.

## 1 Introduction

Each year, millions of travelers and seasonal workers undertake rapid trans-altitudinal ascent (typically exceeding 2,500 meters above sea level) (Tremblay and Ainslie, 2021), generating a critical, yet often overlooked, global public health challenge (Luks and Hackett, 2022). While the resultant hypobaric hypoxia is known to precipitate severe pathologies, such as the canonical manifestations of high altitude pulmonary edema and high altitude cerebral edema (Luks and Hackett, 2022; Gatterer et al., 2024), the differential impact on subtle organ systems remains less resolved. Specifically, in contrast to the heavily investigated cardiorespiratory axis, renal physiology confronts distinct bioenergetic demands (Wang et al., 2022). The kidney, which receives 20–25% of total cardiac output to drive active solute transport (Edwards and Kurtcuoglu, 2022), possesses an intrinsically low medullary pO2 (Schurek, 1988). This renders the nephron highly susceptible to dysoxia.

At high altitude, systemic hypoxemia is compounded by complex vasoactive and hemodynamic shifts (Parati et al., 2018; Duo et al., 2025), generating critical oxygen supply-demand mismatches (Murray and Horscroft, 2017; Avellanas Chavala, 2018). Despite this established physiological susceptibility, a significant translational void persists concerning the temporal dynamics of renal acclimatization. Current investigation disproportionately focuses on either the immediate acute phase (<24 hours post-ascent) (Goldfarb-Rumyantzev and Alper, 2014) or the chronic phase (>6 months) (Arestegui et al., 2011; Zhang L. et al., 2025), thereby overlooking the sub-acute interval (3–28 days). We posit this sub-acute window as a critical inflection point governing the bifurcation between successful functional adaptation and the potential progression toward irreversible structural decline. Under low atmospheric pressure and low oxygen environment, hyperventilation improves systemic oxygenation but necessitates renal compensation for the ensuing respiratory alkalosis (Goldfarb-Rumyantzev and Alper, 2014). The imperative to excrete excess bicarbonate requires complex hemodynamic and tubular transport realignments; under hypoxic conditions, these metabolic adjustments strain the already compromised renal bioenergetics, further aggravating tissue hypoxia (Nourbakhsh and Singh, 2014). Crucially, the relationship between structural integrity and measured filtration function remains largely unverified during the sub-acute transition. Data derived from ischemic kidney models indicate that “silent” microvascular rarefaction—specifically the dropout of PTC and Outer Medullary (OM) capillary congestion—can progress even if conventional functional markers stabilize (Basile et al., 2012; Venkatachalam et al., 2015). If this structural-functional dissociation holds true under hypobaric hypoxia, reliance solely on standard clinical metrics (e.g., CRE) may mask significant underlying microvascular dropout, thus resulting in severe underestimation of chronic renal risk in populations exposed to high altitude.

Given the unique intra-renal oxygen gradient and the immense metabolic demands of the tubulointerstitial compartment, the outer medulla, particularly the thick ascending limb of Henle’s loop, and proximal tubules are intrinsically the most vulnerable targets to systemic hypoxia. Consequently, this study integrates the temporal dynamics of tubulointerstitial injury and microvascular rarefaction with a detailed morphometric analysis of the glomerulus. This approach aims to capture the full spectrum of renal adaptation, ranging from early hemodynamic shifts to secondary structural compensation. To characterize this kinetics, we established a 28-day rat model of hypobaric hypoxia simulating an altitude of 5,000 meters. We correlated functional decline with specific pathological remodeling indices, including detailed quantification of PTC density and medullary vascular congestion. Recognizing that biological adaptation often follows non-linear trajectories, we utilized RCS modeling to capture the time-dependent evolution of renal modifications. Concurrently, exploratory ROC analysis was employed to compare the individual-level discriminatory patterns of predefined circulating biomarkers, including NGAL and KIM-1, against early histological injury. This analysis was intended to provide hypothesis-generating information rather than clinical diagnostic validation. IL-18 was included as an exploratory inflammatory marker to assess whether hypobaric hypoxia was accompanied by a systemic or inflammasome-related inflammatory response in parallel with tubular stress. We hypothesized that renal adaptation to sustained hypobaric hypoxia would show a non-linear temporal pattern, in which early functional changes may diverge from progressive microscopic structural injury.

## 2 Materials and methods

### 2.1 Animals and experimental treatments

All animal experiments were conducted according to the stipulations outlined in the Regulations for the Administration of Affairs Concerning Experimental Animals (2017 Revision) and the Laboratory Animals-General Requirements for Animal Experiment (GB/T 35823-2018). The experimental design was approved by the Experimental Animal Ethics Committee of Beijing Shijitan Hospital, Capital Medical University [approval number: sjtkkll-lx-2023(011); January 17, 2023]. The personnel conducting the experimental operations strictly abide by the 3R principles of animal experiments throughout the entire experimental process. Eight-week-old male SD rats (weight 250 ± 10g) were used for these experiments, which were purchased from Charles River Laboratories Co. Ltd., Beijing, China (certificate number: SCXK 2021-0011). Animals were housed in the Clinical Experimental Animal Center of Beijing Shijitan Hospital. After one week of adaptive feeding, the experiments were carried out. During this period, the animals had free access to food and water. The temperature in the breeding room was (22 ± 2 °C), and the humidity was 50% ± 10%. A 12-hour light and 12-hour dark circadian rhythm was maintained. A total of 30 rats were randomly divided into: control group (6 rats): were maintained under a normobaric normoxic environment for 28 days. Experimental group (24 rats): entered a hypobaric hypoxic chamber (DSF-II animal experimental negative pressure chamber, produced by Weifang Huaxin Oxygen Industry Co., Ltd.), simulating an altitude of 3000 meters (atmospheric pressure 70.1 kPa, PO2: 14.7 kPa) for 2 days, after 2 days of exposure at a simulated altitude of 3000 m, animals were further exposed to a simulated altitude of 5000 m (atmospheric pressure 54.0 kPa, PO2: 11.3 kPa). The experimental group was further randomly divided into 4 groups, living in the simulated 5000 m altitude environment for 3 days, 7 days, 14 days, and 28 days, respectively. A single normoxic control group was maintained under normobaric normoxia for 28 days and was used as the reference group. Separate time-matched normoxic control groups at Day 3, Day 7, and Day 14 were not included. The sample size of n = 6 per group was selected based on experimental feasibility, ethical considerations regarding animal use, and the exploratory nature of this longitudinal histopathological study. No formal *a priori* power calculation was performed.

### 2.2 Haematological and biochemical analysis

After the animals in each experimental group maintained in the hypobaric hypoxia chamber for a set length of time, the rats were anesthetized with sodium pentobarbital, and 2–3 mL of blood was taken from the heart, which was allowed to stand at room temperature for 1h and then centrifuged at 1,500 × *g* for 20 min at 4°C, and the upper layer of serum was stored at −80°C for measurement. Serum was the only biofluid collected in this study; urine samples were not collected. All serum biomarker assays were performed in a single analytical batch according to the manufacturers’ protocols and the operators were blinded.

Serum concentrations of renal biomarkers were quantified using commercially available enzyme-linked immunosorbent assay (ELISA) and colorimetric assay kits, strictly adhering to the manufacturer’s protocols.

Specific kits used were as follows: NGAL (ab119602, Lot: GR3362911-1; Abcam, Cambridge, MA, USA), KIM-1 (ab119597, Lot: GR3413976-2; Abcam), CysC (ab201281, Lot: GR3416876-2; Abcam), and IL-18 (ab213909, Lot: GR3388522-1; Abcam). Serum CRE levels were determined using a Creatinine Assay Kit (ab65340, Lot: GR3405925-2; Abcam) based on the sarcosine oxidase-peroxidase method.

Briefly, serum samples were thawed on ice and centrifuged at 1500 × *g* for 10 minutes to remove particulates. Standards and samples were pipetted into 96-well microplates pre-coated with specific capture antibodies. Following incubation and washing steps, the appropriate biotinylated detector antibody and HRP-streptavidin conjugate were added. The reaction was developed with TMB substrate and stopped with an acidic solution. Optical density was measured at 450 nm (for ELISA kits) or 570 nm (for the creatinine assay) using a UV-2600 Spectrophotometer (Sunny Hengping Instrument, Shanghai, China). Concentrations were calculated by interpolation from a standard curve generated for each plate using 4-parameter logistic regression analysis. Assay quality-control data, including duplicate-well coefficient-of-variation calculations where available and biological CV summaries, are provided in **Supplementary Table 4**.

### 2.3 Renal pathology processing

At the designated experimental endpoint, rats were anesthetized with sodium pentobarbital as described above. Blood was collected by cardiac puncture before renal tissue harvesting. After terminal blood collection, the abdominal cavity was opened through a midline incision, and both kidneys were exposed. The right kidney was excised first, the renal capsule was carefully removed, and surface blood was gently blotted without compression of the renal parenchyma. Right kidney weight was measured using an electronic balance and recorded in grams to two decimal places. Kidney length was defined as the maximal longitudinal distance from the upper pole to the lower pole and was measured using a caliper. The right kidney was then cut coronally to include the cortex, corticomedullary junction, and outer medulla. Tissue samples were immersion-fixed in 10% neutral-buffered formalin for 24 h at room temperature, processed routinely, embedded in paraffin, and used for H&E, PAS, and immunohistochemical staining. No transcardial or renal arterial perfusion with saline or fixative was performed before kidney removal. The left kidney was snap-frozen in liquid nitrogen and stored at −80 °C for potential biochemical analyses. Therefore, all histological and morphometric analyses reported in this study were performed on the right kidney to maintain sampling consistency across animals.

Paraffin-embedded renal tissues were sectioned at a thickness of 4 μm. For H&E and PAS staining, sections were deparaffinized in xylene, rehydrated through a graded ethanol series, rinsed in distilled water, and stained according to standard protocols. H&E-stained sections were used to assess general renal morphology, tubular injury, and outer medullary erythrocyte accumulation/congestion-like changes. PAS-stained sections were used to evaluate tubular brush border integrity, tubular luminal changes, and glomerular morphology. Whole-slide images were acquired using a DX12 (3DHISTECH SHANDONG, Shandong, China) at 40× equivalent magnification and representative images were reviewed using CaseViewer 2.4 (3DHISTECH Ltd., Budapest, Hungary). Morphometric measurements were performed using Zeiss Digital Pathology Software ZEN 3.3.

To assess renal microvascular integrity, PTC density was visualized using immunohistochemical staining for CD34, a specific marker of vascular endothelial cells. Paraffin-embedded renal tissues were sectioned at a thickness of 4 μm. Sections were deparaffinized in xylene and rehydrated through a graded ethanol series. Antigen retrieval was performed by microwave heating in citrate buffer (pH 6.0) for 15 min to unmask epitopes. To block endogenous peroxidase activity, sections were incubated with 3% hydrogen peroxide for 15 min. Subsequently, non-specific binding sites were blocked by incubation with 5% bovine serum albumin for 30 min at room temperature. The sections were then incubated overnight at 4 °C with a primary rabbit polyclonal anti-CD34 antibody (1:100 dilution; ab81289, Abcam, Cambridge, MA, USA). Following three washes with phosphate-buffered saline, sections were incubated with a horseradish peroxidase-conjugated goat anti-rabbit secondary antibody for 1 h at room temperature. Immunoreactivity was visualized using a 3,3′-diaminobenzidine substrate kit, resulting in brown staining of endothelial cells. Nuclei were counterstained with hematoxylin, and sections were dehydrated, cleared, and mounted.

Ten high-power fields (200×) at the corticomedullary junction were randomly selected from each kidney section for semi-quantitative assessment of renal tubular injury and medullary vascular congestion. Renal tubular injury was evaluated using a semi-quantitative RTI score adapted from previously published criteria (Zhang et al., 2018). This score was intended to capture the overall severity of hypoxia-associated tubular epithelial and luminal alterations, rather than to classify lesions strictly as acute or chronic tubular injury. The assessed features included tubular luminal narrowing or dilation, epithelial cell swelling, epithelial atrophy or loss, and reduction in tubular epithelial cell number. Each field was scored on a 0–5 scale according to the proportion of affected tubules: 0, no obvious abnormality; 1, <10%; 2, 10–25%; 3, 25–50%; 4, 50–75%; and 5, >75%. The mean score from ten fields was used for each animal. All scoring was performed in a blinded manner using the same criteria across all groups. For outer medullary erythrocyte accumulation/congestion-like changes, the outer medulla region was assessed on H&E-stained sections using a 0–5 scale according to the estimated proportion of congested visible vessels: 0, no congestion; 1, approximately 20%; 2, approximately 40%; 3, approximately 60%; 4, approximately 80%; and 5, approximately 100% congestion (McLarnon et al., 2022). Because renal tissues were not perfusion-fixed before removal, this score was used as a descriptive morphological index and was interpreted cautiously. CD34-defined peritubular capillary (PTC) density scoring: 10 areas of 120,000 um^2^ were circled per section, and the number of PTCs/number of renal tubules (PTC/tubule) ratio was calculated in that area (Steegh et al., 2011; van der Weijden et al., 2023). Measurement of glomerular size: direct measurement using glomerular capillary tuft in the cortical as follows (Wang et al., 2013; Grande et al., 2021): two longest diameters of the glomerular capillary tuft on the largest sectioned glomeruli [glomeruli containing vascular and/or urinary poles (pole-containing glomeruli)] in pathologic photographs were measured perpendicularly to each other using the Zeiss Digital Pathology Software (ZEN 3.3), and averaged. Ten glomeruli were measured in each rat kidney section, and differences in glomerular size were compared between groups. All of the above scores were performed using a blinded method.

### 2.4 Statistical analysis

Statistical analyses were performed using SPSS version 22.0 (IBM Corp., Armonk, NY, USA), R software version 4.4.2 (The R Foundation for Statistical Computing, Vienna, Austria) and MSTATA software (https://www.mstata.com/). GraphPad Prism 8 (GraphPad Software, San Diego, CA, USA) was utilized for initial data visualization. Continuous variables were first assessed for normality using the Shapiro-Wilk test. Data conforming to a normal distribution are presented as mean ± standard deviation (SD). Non-normally distributed data are expressed as median with interquartile range (IQR, 25th–75th percentiles). Categorical data are reported as frequencies or percentages. Normally distributed variables were analyzed using one-way analysis of variance (ANOVA) followed by Tukey’s *post hoc* test for pairwise comparisons. For acute stress and functional serum biomarkers, descriptive statistics are reported as mean ± SD and median [IQR] for each group. Overall test statistics, including the ANOVA F statistic, degrees of freedom, and P value, are provided in the supplementary statistical table. Tukey-adjusted *post hoc* P values were used to determine pairwise differences between time points. Unadjusted P values were not used to assign statistical significance for pairwise comparisons. Non-normally distributed or ordinal data were analyzed using the Kruskal–Wallis test with Bonferroni-adjusted *post hoc* comparisons. For group comparisons, the normoxic control group was used as the reference group and is referred to as ‘Control’ throughout the revised manuscript.

An exploratory receiver operating characteristic (ROC) analysis was performed to compare the individual-level discriminatory performance of a predefined biomarker panel for early histological renal injury at Day 3 of hypoxic exposure. This analysis was not intended to repeat group-comparison testing between Day 3 and Control animals, but rather to examine whether each circulating marker could discriminate animals with early histological tubular injury from non-injured animals. Early renal injury was defined *a priori* as an RTI score ≥ 2 at Day 3, based on blinded histopathological assessment. Animals from the normoxic control group were classified as non-injured. ROC curves were generated for KIM-1, NGAL, CysC, and CRE measured at Day 3. The AUC was calculated to quantify discriminatory ability, and 95% confidence intervals (95% CIs) for AUCs were estimated using the DeLong method. Pairwise comparisons between AUCs were performed using DeLong’s test. Because multiple biomarker AUCs were compared, DeLong pairwise P values were adjusted using Tukey correction. Adjusted P < 0.05 was considered statistically significant for pairwise AUC comparisons. For exploratory purposes, the Youden index was calculated to identify optimal cut-off values for each biomarker. Given the small sample size and the exploratory design, these ROC analyses were interpreted as hypothesis-generating and not as clinical diagnostic validation.

To explore the associations between structural damage and functional markers, Spearman’s rank correlation analysis was performed. A correlation matrix heatmap was generated using the R package ‘corrplot’. To visualize the temporal hierarchy of renal alterations, a Standardized Rate-of-Change Heatmap was constructed. All parameters were included in the rate-of-change heatmap to focus on dynamic changes. Rates of change were calculated as relative percentage change per day between adjacent time points. The daily rate of change for each marker was calculated as: *Rate* = [(*Value_t_*_2_ – *Value_t_*_1_)/*Value_t_*_1_]/(*t*2-*t*1) × 100%. Although pathological scores (RTI score and OM congestion score) are ordinal, rate-of-change analysis was applied to visualize temporal progression trends rather than absolute quantitative change; for these ordinal variables, absolute change per day was calculated as: *Rate* = (*Value_t_*_2_ – *Value_t_*_1_)/(*t*2-*t*1) × 100%. To explore potential non-linear associations between the duration of hypoxic exposure and renal markers, RCS functions were fitted within a linear regression framework using MSTATA software. The exposure duration was treated as a continuous variable, and four knots were placed at the 5th, 35th, 65th, and 95th percentiles of its distribution. The overall association between exposure duration and renal markers was assessed using the global test (*P-overall*), while non-linearity was evaluated using the non-linear component test (*P-nonlinear*). An inflection point was further identified based on the fitted spline curves to characterize changes in the trajectory of the association. A two-tailed *P-value* < 0.05 was considered statistically significant for all analyses.

For histological and morphometric parameters, the animal was used as the unit of statistical analysis. Multiple glomeruli or microscopic fields measured within the same kidney were treated as within-animal technical or sampling replicates and were averaged to obtain one biological value per animal before group-level statistical testing.

## 3 Results

### 3.1 Macroscopic renal characteristics

**Figure 1:**
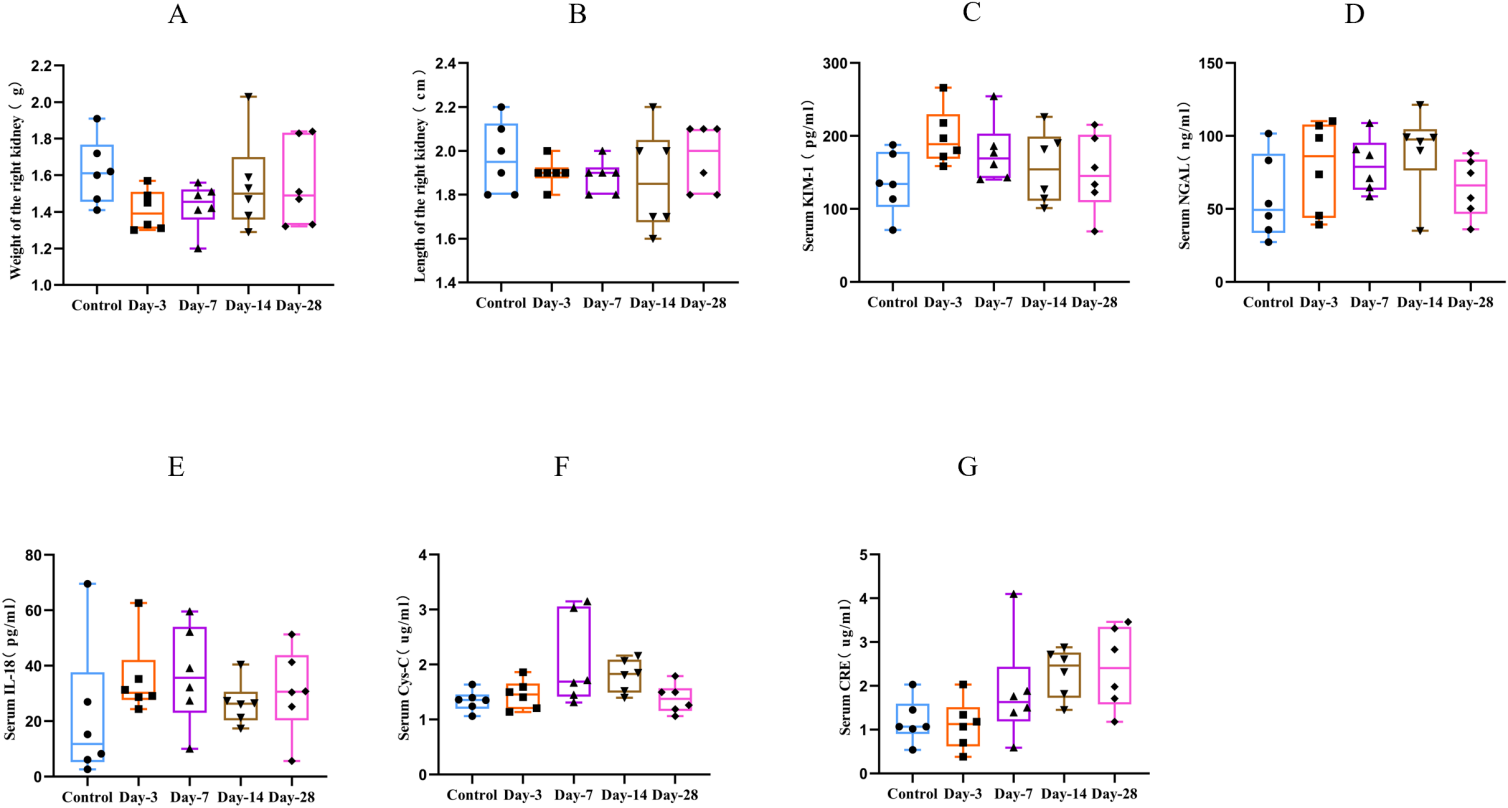
Macroscopic and biochemical characteristics of hypoxic renal injury. Box-and-scatter plots showing individual data points (N = 6 per group), medians, and interquartile ranges. **(A)** Right Kidney Weight, **(B)** Right Kidney Length, **(C)** Kidney injury molecule-1(KIM-1), **(D)** Neutrophil gelatinase-associated lipocalin (NGAL), **(E)** Serum interleukin-18 (IL-18), **(F)** Cystatin C (CysC), and **(G)** Serum creatinine (CRE). Overall group differences were assessed using one-way ANOVA followed by Tukey-adjusted *post hoc* comparisons for pairwise testing. No individual pairwise comparison among the serum biomarkers reached statistical significance after Tukey correction. Detailed descriptive statistics and Tukey-adjusted P values are provided in **Supplementary Table 1**.

To evaluate the gross anatomical impact of high-altitude exposure, we analyzed the changes in right kidney weight and length. Box-and-scatter plots (**Figures 1A, B**) illustrated the distribution of these macroscopic parameters across all time points. The plots revealed no statistically significant differences in absolute right kidney weight (P = 0.291) or right kidney length (P = 0.675) among the control and hypoxic groups. Because body weight was not consistently recorded across all groups, kidney weight could not be normalized to body weight; therefore, these macroscopic parameters were interpreted as descriptive gross anatomical indices.

### 3.2 Temporal dynamics of serum biomarkers

We further tracked the kinetic profiles of five serum biomarkers using box-and-scatter plots (**Figures 1C–G**). Descriptive statistics, including mean ± SD, median [IQR], biological CV, overall test statistics, and Tukey-adjusted pairwise comparisons, are provided in **Supplementary Table 1**.

For acute tubular stress and inflammatory markers, serum KIM-1, NGAL, and IL-18 (**Figures 1C–E**) showed variable temporal patterns across the exposure period. However, overall ANOVA did not demonstrate statistically significant temporal differences for KIM-1, NGAL, or IL-18. Tukey-adjusted *post hoc* comparisons also did not identify significant pairwise differences between individual time points. Therefore, these markers were interpreted as exploratory temporal readouts rather than definitive time-point-specific changes.

For filtration-related markers, overall ANOVA suggested temporal variation for CysC and CRE (**Figures 1F, G**). CysC showed a numerical increase around Day 7 followed by a decline at later time points, whereas CRE showed a gradual numerical increase during prolonged hypoxic exposure. However, Tukey-adjusted *post hoc* comparisons did not identify statistically significant pairwise differences between individual time points. These serum biomarker trajectories should therefore be interpreted cautiously, particularly given the small sample size and inter-animal heterogeneity.

### 3.3 Exploratory individual-level discrimination of early histological injury at Day 3

**Figure 2:**
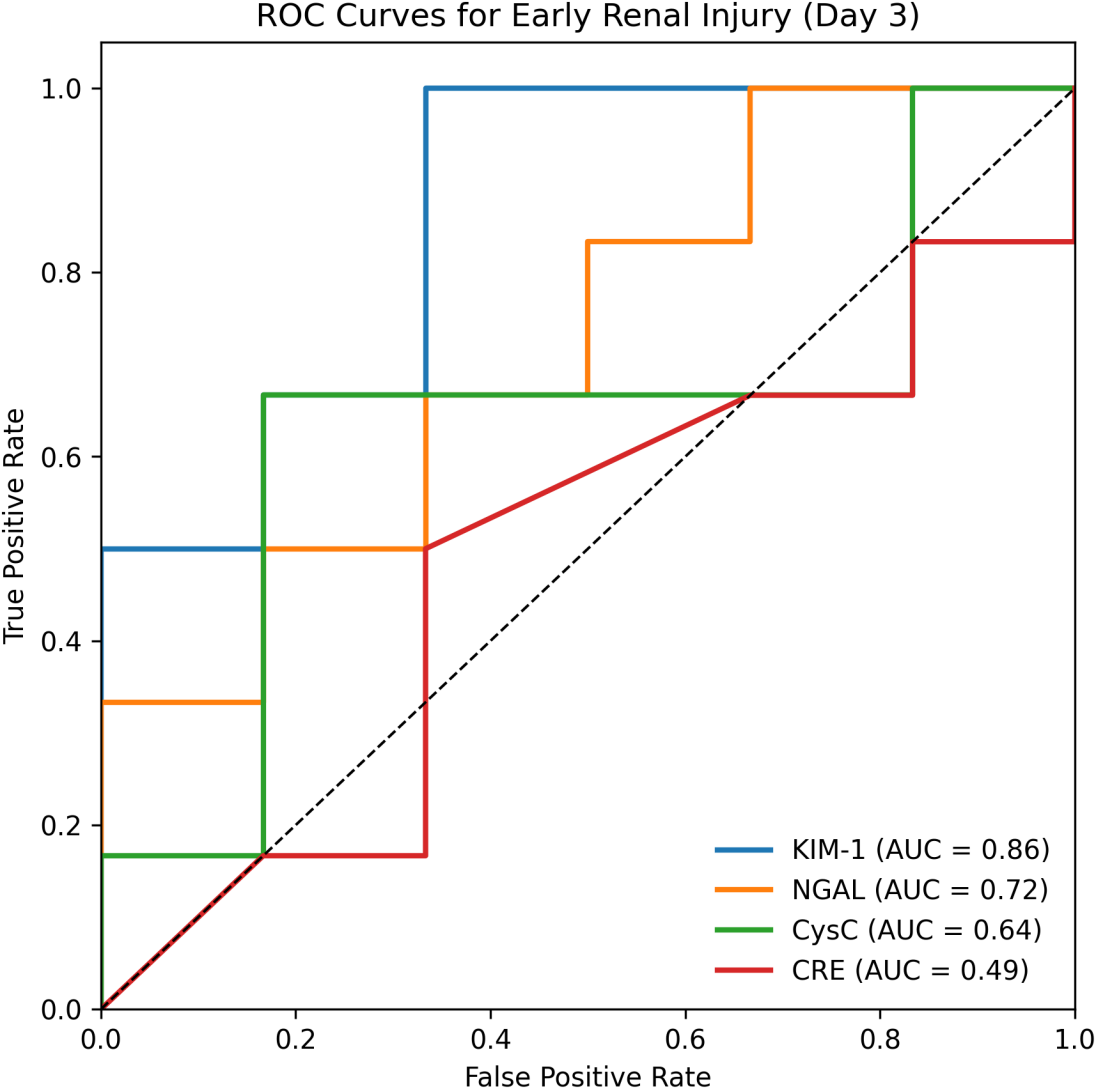
Exploratory ROC analysis of predefined circulating biomarkers for early histological renal injury at Day 3. Early injury was defined *a priori* as RTI score ≥ 2. KIM-1, NGAL, CysC, and CRE were included as a predefined biomarker panel. The normoxic Control group was used as the non-injured reference group, and early hypoxic injury was assessed in animals exposed to hypobaric hypoxia for 3 days. AUC values are shown with 95% confidence intervals estimated using the DeLong method. Pairwise AUC comparisons were performed using DeLong’s test, followed by Tukey correction for multiple comparisons. No pairwise AUC comparison remained statistically significant after Tukey correction. Therefore, the ROC analysis should be interpreted as exploratory and hypothesis-generating rather than as clinical diagnostic validation.

To explore whether circulating biomarkers could discriminate early histological renal injury at the individual-animal level, an exploratory ROC analysis was performed at Day 3 using RTI score ≥ 2 as the predefined histological reference standard. The corresponding ROC curves and AUC comparisons are shown in **Figure 2**. This analysis was performed as a complementary assessment to group-comparison testing and was not intended to imply that all examined markers showed significant mean differences from the Control group at Day 3. Because Tukey-adjusted comparisons did not confirm significant time-point-specific differences among the serum biomarkers, the ROC analysis was interpreted as a complementary exploratory assessment of individual-level discrimination rather than as evidence of validated diagnostic performance. Given the small sample size and the absence of significant Tukey-adjusted pairwise differences among serum biomarkers in group-level analyses, this ROC analysis was considered complementary and exploratory.

Among the predefined biomarkers, KIM-1 showed the highest exploratory AUC (AUC = 0.861, 95% CI: 0.641–1.000), followed by NGAL (AUC = 0.722, 95% CI: 0.414–1.000), CysC (AUC = 0.639, 95% CI: 0.273–1.000), and CRE (AUC = 0.486, 95% CI: 0.124–0.848). These wide confidence intervals indicate substantial uncertainty in the AUC estimates, consistent with the limited sample size.

Pairwise comparisons of AUCs were performed using DeLong’s test, followed by Tukey correction for multiple comparisons. No pairwise AUC comparison remained statistically significant after Tukey correction. The comparison between KIM-1 and CRE showed the largest separation before correction (raw DeLong *P = 0.0204*), but this difference did not remain statistically significant after Tukey correction (adjusted *P = 0.0938*). Therefore, the ROC findings should be interpreted as exploratory and hypothesis-generating rather than as evidence of validated diagnostic superiority. Detailed AUC estimates, 95% CIs, and DeLong/Tukey-adjusted pairwise comparisons are provided in **Supplementary Table 2**.

Cutoff-based performance metrics were accompanied by 95% confidence intervals because of the small number of animals included in the Day 3 ROC analysis. KIM-1 showed the highest exploratory sensitivity at the Youden-derived cutoff, but the corresponding confidence intervals were wide. Therefore, sensitivity, specificity, PPV, NPV, accuracy, and cutoff values should be interpreted cautiously as exploratory estimates. Detailed performance indices and 95% confidence intervals are provided in **Supplementary Table 3**.

### 3.4 Histopathological progression and microvascular rarefaction

**Figure 3:**
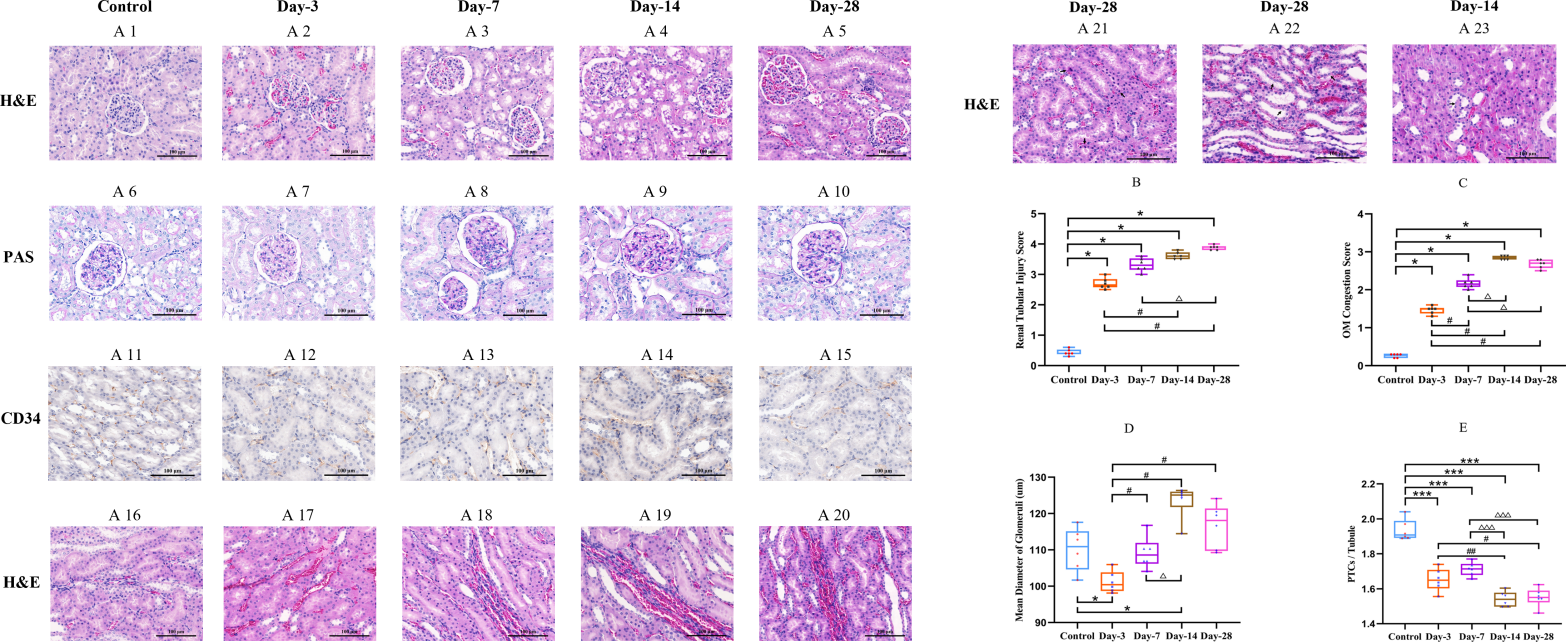
Temporal Evolution of Renal Histopathology and Quantitative Morphometry under Hypobaric Hypoxia. **(A)** Representative photomicrographs of renal tissue sections arranged by staining method (rows) and duration of hypoxic exposure (columns: Control, 3, 7, 14, and 28). Original magnification: 200×; Scale bar = 100 μm. **(A1–A5)** H&E staining of the renal cortex. **(A6–A10)** PAS staining visualizing tubular basement membranes and brush borders. **(A11–A15)** CD34 immunohistochemistry identifying vascular endothelial cells of peritubular capillaries (positive staining appears brown). **(A16–A20)** H&E staining of the outer medulla, focusing on the vascular bundles (vasa recta). **(A21)** H&E staining from the day 28 group showing diffuse tubular epithelial injury, characterized by epithelial cell swelling, increased eosinophilia or cytoplasmic clouding, nuclear hyperchromasia with pyknotic changes, and focal tubular architectural distortion with irregular luminal contours and disorganized epithelial alignment (black arrows). **(A22)** H&E staining from the day 28 group demonstrating marked tubular epithelial swelling with diffuse cytoplasmic vacuolar degeneration, predominantly involving proximal tubules (black arrows). **(A23)** H&E staining from the day 14 group showing focal sloughing of tubular epithelial cells into the tubular lumen (black arrows). **(B-E)** Quantitative Morphometry: Box-and-scatter plots showing the statistical analysis of structural remodeling (n=6 per group): **(B)** Tubular Injury Score, **(C)** Outer Medulla Congestion Score, **(D)** Glomerular Diameter, and **(E)** PTC/tubule ratio. Each dot represents one animal-level value. For glomerular diameter, each dot represents the mean value of ten glomeruli measured from one rat. For PTC/tubule ratio, each dot represents the mean value of ten quantified fields from one rat. For RTI and outer medullary erythrocyte accumulation/congestion-like scores, ten fields were scored per animal and averaged to generate one animal-level score. Group comparisons were performed using n=6 animals per group. Individual glomeruli or microscopic fields were not treated as independent biological replicates. *P < 0.05, **P < 0.01, ***P < 0.001 vs. Control; ^#^P < 0.05, ^##^P < 0.01, ^###^P < 0.001 vs. Day 3; ^△^P < 0.05, ^△△^P < 0.01, ^△△△^P < 0.001 vs. Day 7.

Histological examination revealed distinct temporal patterns of renal injury (**Figure 3A**). In the Control group, renal architecture was preserved, with intact glomeruli, regularly arranged tubules, patent peritubular capillaries, and no evident medullary congestion. In the hypoxia-exposed groups, time-dependent structural alterations were observed. Mean glomerular diameter showed a biphasic pattern, with an initial diameter reduction at Day 3, followed by a progressive increase from Day 7 and a peak at Day 14. Tubulointerstitial changes included tubular luminal narrowing or dilation, epithelial cell swelling, vacuolar degeneration, and focal epithelial detachment. In the outer medulla, increased intravascular erythrocyte accumulation and congestion-like changes were observed with prolonged hypoxic exposure. Because kidneys were not perfusion-fixed before removal, this parameter was interpreted as a semi-quantitative morphological indicator rather than as direct evidence of *in vivo* medullary blood-flow stasis.

Quantitative analyses using box-and-scatter plots (**Figures 3B–E**) further confirmed these temporal changes. Tubular injury scores and outer medullary congestion scores increased significantly over time and reached higher levels at Days 14–28 (*P* < 0.001). Mean glomerular diameter was significantly reduced at Day 3 compared with the control group and subsequently increased, reaching a level above Control at Day 14 (*P* < 0.05). *Post hoc* pairwise comparisons also showed significant differences between Day 3 and later hypoxia-exposure time points, supporting a transition from early glomerular diameter reduction to subsequent enlargement. Day 3 was not treated as a control group in these analyses, but was included as a comparison time point to characterize the biphasic temporal pattern. In contrast, the CD34-defined peritubular capillary-to-tubule (PTC/tubule) ratio declined significantly from Day 3 onward and remained reduced at later time points (*P* < 0.001).

### 3.5 Standardized temporal dynamics of renal alterations

**Figure 4:**
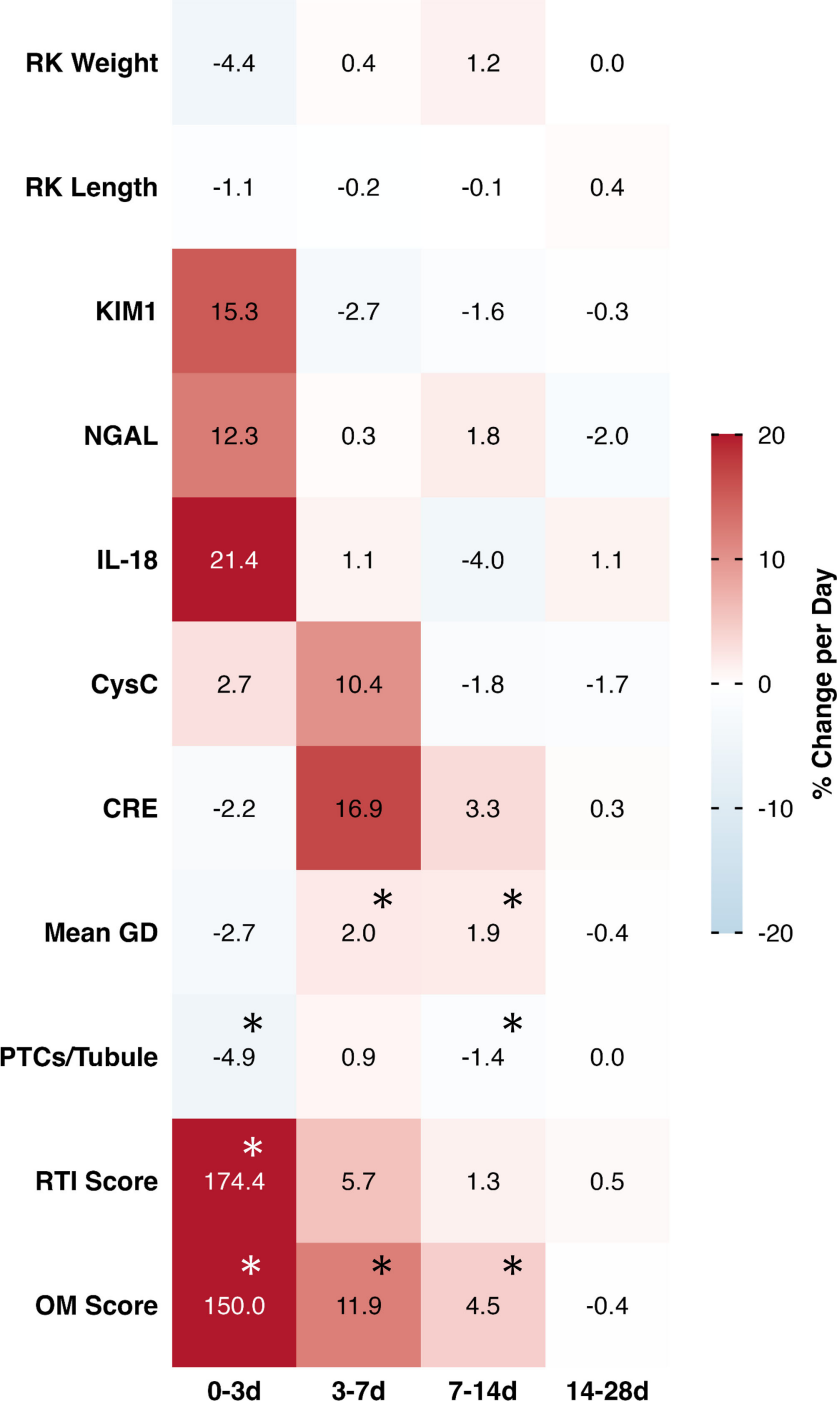
Standardized temporal rate-of-change heatmap of renal parameters during hypobaric hypoxia exposure. The heatmap visualizes the daily rate of change of renal indicators across four adjacent time intervals: Days 0–3, 3–7, 7–14, and 14–28. For continuous variables, rates of change were calculated as the relative percentage change per day between adjacent time points. For ordinal pathological scores, including the renal tubular injury score and outer medullary congestion-like score, changes were expressed as absolute change per day to improve interpretability, particularly in the presence of zero or near-zero baseline values. Color intensity represents the magnitude and direction of change, with red indicating an increase and blue indicating a decrease. All parameters were retained in the heatmap to visualize the temporal hierarchy and directionality of changes across functional, structural, and pathological domains. Asterisks indicate intervals showing statistically significant differences between adjacent time points after post hoc correction (adjusted P < 0.05). Significant intervals were observed for mean glomerular diameter during Days 3–7 and 7–14, PTCs/tubule during Days 0–3 and 7–14, renal tubular injury score during Days 0–3, and outer medullary congestion-like score during Days 0–3, 3–7, and 7–14. For pathological and morphometric variables, animal-level mean values were used for statistical testing; ten measurements or microscopic fields were first averaged within each rat, and group comparisons were then performed using n = 6 animals per group. This heatmap is intended as a descriptive visualization of temporal dynamics and should not be interpreted as an independent causal analysis.

To compare the temporal dynamics of renal alterations across molecular, structural, and functional domains, a rate-of-change heatmap was constructed (**Figure 4**), normalizing the magnitude of change across consecutive time intervals. Distinct temporal patterns were observed among injury-related parameters.

During the early interval (Day 0–3), RTI score, OM congestion-like score, and serum KIM-1 showed the greatest positive rates of change (+174.4%/day, +150.0%/day, and +15.3%/day, respectively), coinciding with the most pronounced decline in the PTC/tubule ratio (−4.9%/day). However, statistically significant adjacent-interval changes after *post hoc* correction were observed primarily for pathological and morphometric variables, rather than for serum biomarkers.

In the subsequent interval (Day 3–7), CRE and CysC showed the largest numerical rates (+16.9%/day and +10.4%/day, respectively) of change among functional biomarkers. These changes were retained in the heatmap to visualize directionality and temporal hierarchy, but they did not correspond to statistically significant Tukey-adjusted pairwise differences.

During later intervals (Day 14–28), the rates of change for most injury-related parameters decreased substantially, approaching zero.

### 3.6 Temporal patterns of functional biomarkers and histological injury

**Figure 5:**
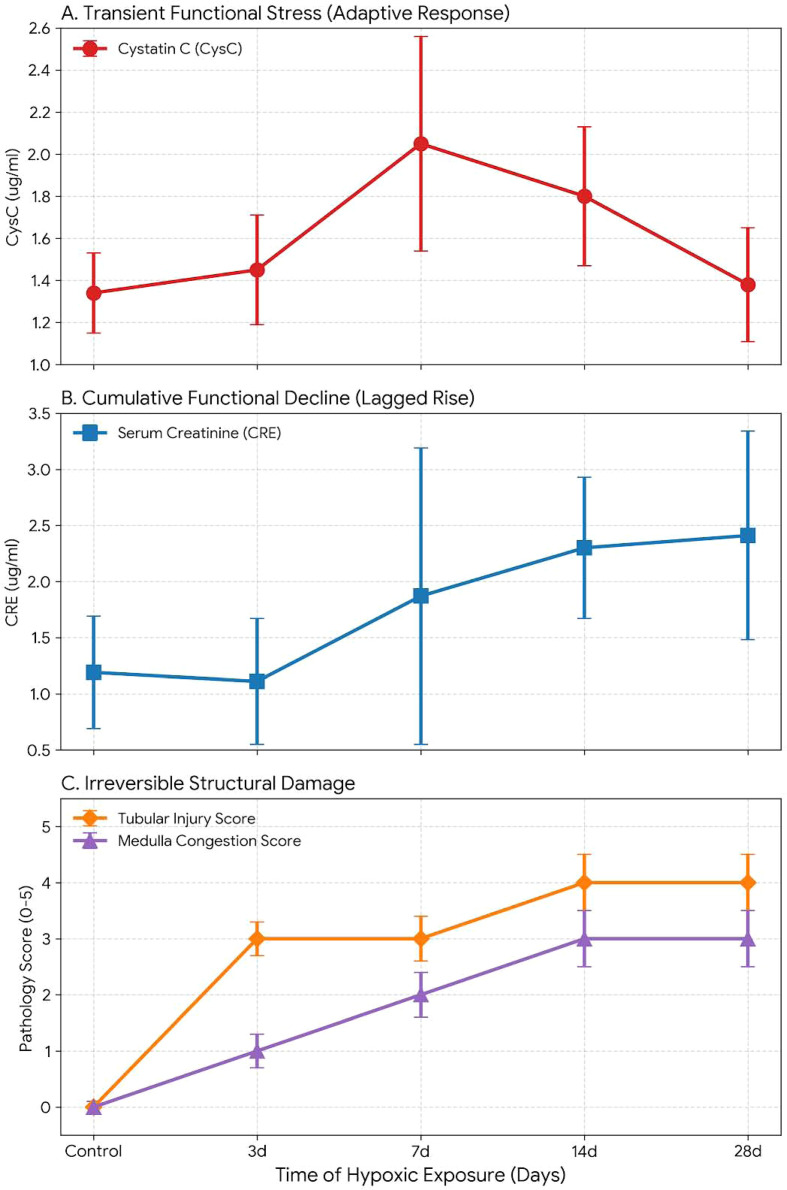
Integrated temporal comparison of filtration-related biomarkers and histological injury scores during hypobaric hypoxia exposure. **(A)** Temporal changes in serum Cystatin C (CysC) levels. **(B)** Temporal changes in serum creatinine (CRE) levels. **(C)** Longitudinal progression of histological scores, including the RTI score and the OM congestion score. The figure does not present an independent dataset but reorganizes selected functional and histological parameters on a common exposure timeline to visualize their temporal divergence. Serum CysC and CRE are shown as filtration-related functional markers, whereas RTI score and outer medullary congestion-like score are shown as histological injury indices. Data are presented as mean ± SD for biomarkers and median [IQR] for pathological scores. For serum biomarkers, Tukey-adjusted post hoc comparisons did not identify significant pairwise differences between individual time points; therefore, the biomarker trajectories should be interpreted as exploratory temporal patterns.

**Figure 5** was generated as an integrative temporal display rather than as an independent dataset. Whereas **Figure 1** summarizes the full serum biomarker panel and macroscopic renal parameters, and **Figure 2** evaluates exploratory Day 3 biomarker discrimination, **Figure 5** directly juxtaposes filtration-related markers with histological injury scores on the same exposure timeline to visualize the divergence between functional changes and persistent structural injury.

Serum CysC levels (**Figure 5A**) showed a numerical increase around Day 7 followed by a gradual decline toward baseline levels by Day 28. CRE levels (**Figure 5B**) showed a delayed numerical increase, with the highest mean value observed at Day 28. However, Tukey-adjusted *post hoc* comparisons did not confirm statistically significant pairwise differences between individual time points for either marker. Therefore, **Figure 5** should be interpreted as an integrative visualization of temporal patterns rather than as evidence of definitive time-point-specific functional biomarker changes.

Histological scoring demonstrated sustained structural injury throughout the experimental period (**Figure 5C**). The renal tubular injury score increased markedly at Day 3 and remained elevated through Day 28. Similarly, the outer medullary congestion-like score increased from Day 3 and remained higher during prolonged hypoxic exposure. This integrated visualization highlights the temporal dissociation between functional readouts and persistent histological injury during sustained hypobaric hypoxia.

### 3.7 Correlation analysis of renal pathology, morphometry, and function

**Figure 6:**
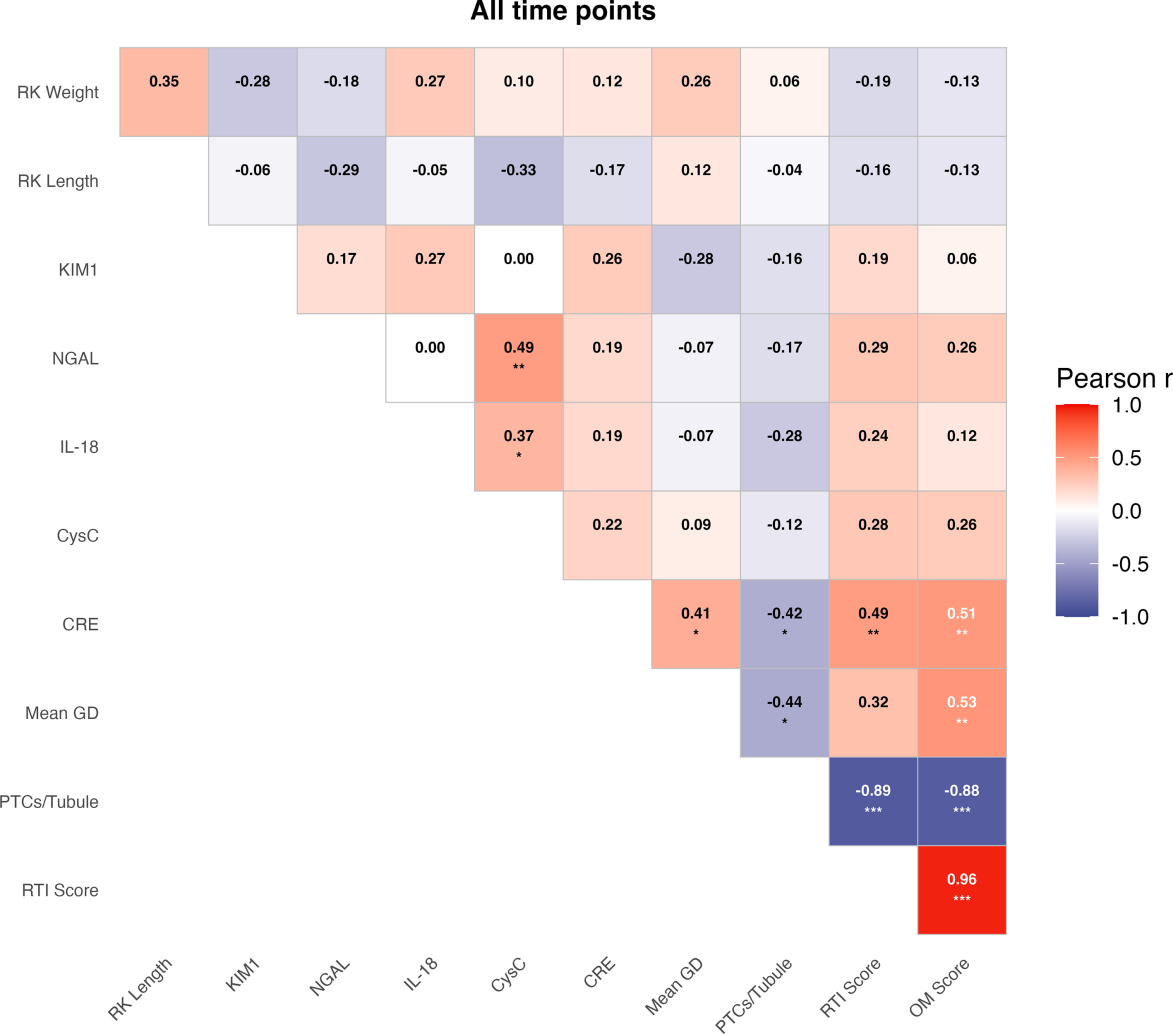
Spearman correlation analysis of renal functional, molecular, and structural parameters across all time points. Spearman’s rank correlation coefficients were calculated to assess monotonic associations among renal injury–related biomarkers, microvascular indices, and histopathological parameters across all experimental time points. ρ denotes Spearman’s rank correlation coefficient. Correlation coefficients are displayed as color-coded heatmaps, with red indicating positive correlations and blue indicating negative correlations. Statistical significance is indicated as follows: *P < 0.05, **P < 0.01, and ***P < 0.001. Abbreviations: Weight, Right kidney weight. Length, Right kidney length; KIM1, Kidney injury molecule-1; NGAL, Neutrophil gelatinase-associated lipocalin; IL18, Interleukin-18; CysC, cystatin C; CRE, serum creatinine; PTCs/Tubule, Peritubular capillaries per tubule; RTI Score, Renal tubular injury score; Mean GD, Mean glomerular diameter; OM Score, Outer medullary congestion score.

Spearman correlation analysis was performed to evaluate associations among molecular biomarkers, microvascular indices, and histopathological parameters across all experimental groups and time points (**Figure 6**). Because this analysis pooled animals across time points, some associations may partly reflect shared temporal trends rather than direct pairwise biological coupling. Significant correlations were observed, with distinct patterns across molecular, vascular, and structural domains.

Among molecular biomarkers, NGAL showed a moderate positive correlation with CysC (*ρ* = 0.49, *P* < 0.01), and IL-18 was also positively correlated with CysC (*ρ* = 0.37, *P* < 0.05). In contrast, no significant correlation was detected between KIM-1 and NGAL.

Peritubular capillary density, expressed as the PTCs/tubule ratio, was inversely correlated with multiple injury-related parameters, including CRE (*ρ* = −0.42, *P* < 0.05), and RTI score (*ρ* = −0.89, *P* < 0.001). The PTCs/tubule ratio was also negatively correlated with the OM congestion score (*ρ* = −0.88, *P* < 0.001).

Strong positive correlations were observed among histopathological measures. RTI score was positively correlated with OM congestion score (*ρ* = 0.96, *P* < 0.001) and with CRE (*ρ* = 0.49, *P* < 0.01). In addition, mean glomerular diameter was positively correlated with OM congestion score (*ρ* = 0.53, *P* < 0.01).

### 3.8 Temporal associations between hypoxic exposure duration and renal injury assessed by restricted cubic spline analysis

**Figure 7:**
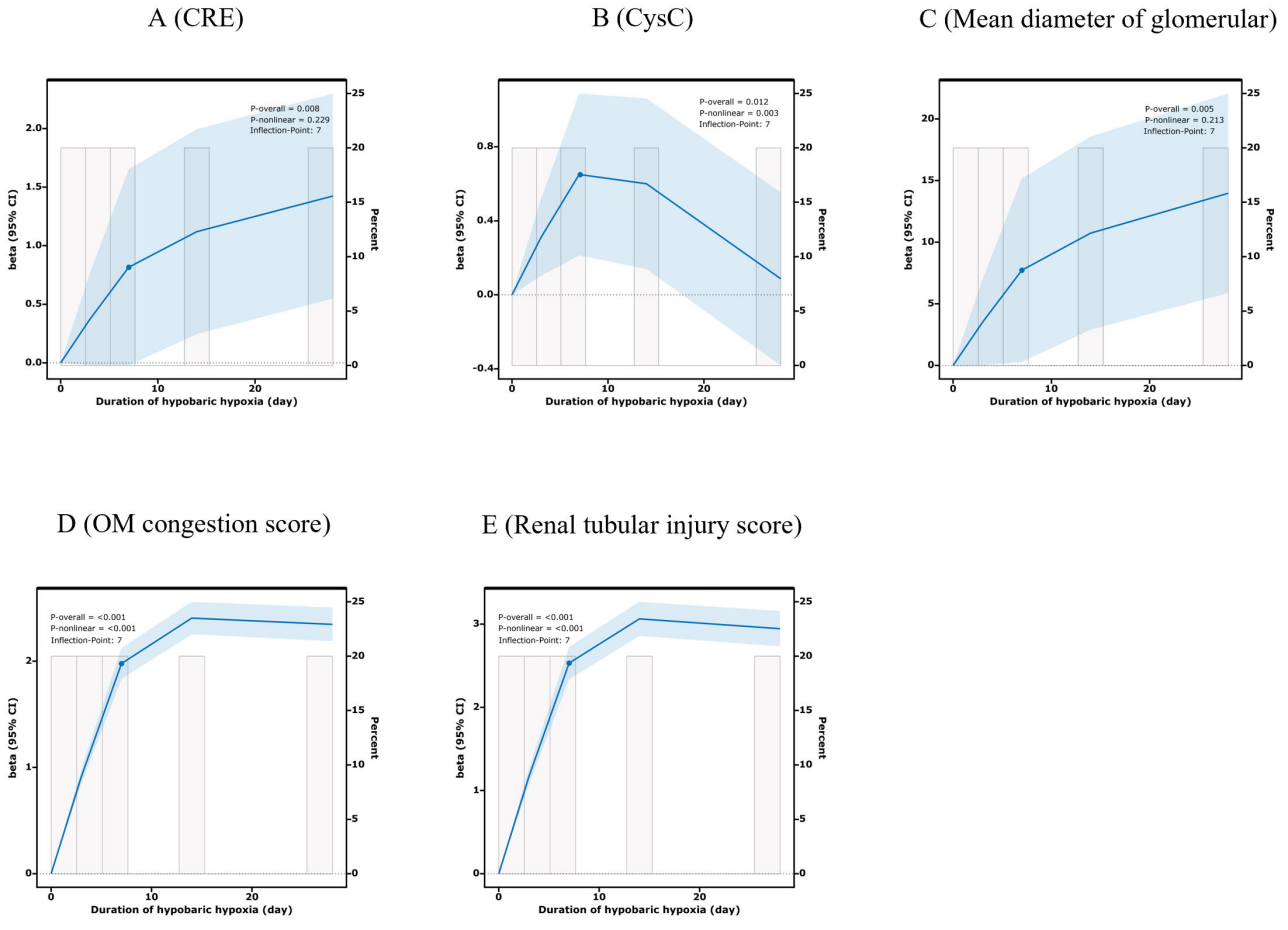
Restricted cubic spline (RCS) analysis of the association between the duration of high-altitude exposure and renal structural and functional indicators. Non-linear relationships were modeled using restricted cubic splines with four knots placed at the 5th, 35th, 65th, and 95th percentiles of exposure duration. In each panel, the solid line represents the estimated regression coefficient (β) relative to the reference value (10th percentile), and the shaded area denotes the 95% confidence interval (95% CI). The right y-axis indicates the corresponding percentage change derived from the β estimates for ease of interpretation. **(A)** Serum creatinine (CRE). **(B)** Serum cystatin C (CysC). **(C)** Mean diameter of glomerular. **(D)** Outer medulla (OM) congestion score. **(E)** Renal tubular injury score. Across panels, the fitted curves suggested an approximate change in slope around Day 7. Given the limited number of experimental time points, this pattern should be interpreted as hypothesis-generating rather than as a definitive biological threshold. Significant non-linearity was observed for panels B, D, and E (P for non-linearity < 0.05).

RCS regression was used to examine the temporal associations between the duration of hypoxic exposure and renal functional and structural indicators (**Figure 7**). For CRE, a statistically significant overall association with exposure duration was observed (*P-overall* = 0.008), whereas the test for non-linearity was not significant (*P-nonlinear* = 0.229), indicating a predominantly linear relationship across the exposure period (**Figure 7A**). In contrast, CysC demonstrated both a significant overall association (*P-overall* = 0.012) and a significant non-linear relationship with exposure duration (*P-nonlinear* = 0.003) (**Figure 7B**). For glomerular mean diameter, a statistically significant overall association with exposure duration was observed (*P-overall* = 0.005), whereas the test for non-linearity was not significant (*P-nonlinear* = 0.213) (**Figure 7C**). Because Tukey-adjusted pairwise comparisons did not identify significant differences between individual time points for serum biomarkers, the RCS findings for CRE and CysC were interpreted as modeled temporal associations rather than definitive evidence of specific between-group differences at individual exposure durations.

For histopathological indices, both OM congestion score and RTI score exhibited highly significant overall associations with hypoxic exposure duration (both *P-overall* < 0.001), as well as pronounced non-linear relationships (both *P-nonlinear* < 0.001) (**Figures 7D, E**). For parameters with significant non-linearity, the fitted spline curves showed a change in slope at approximately Day 7. Before this time point, the estimated slopes increased steeply, indicating rapid changes in structural injury indices. After Day 7, the slopes became attenuated, suggesting a transition toward slower progression or relative stabilization during prolonged exposure. Given the limited number of experimental time points, this Day 7 inflection should be interpreted as a hypothesis-generating temporal pattern rather than a definitive biological threshold.

## 4 Discussion

### 4.1 Overview of principal findings

In this study, we established a rat model of hypobaric hypoxia to systematically map the temporal trajectories of renal acclimatization over a 28-day exposure. By integrating histopathology, biomarker profiling, and RCS modeling, we elucidated the dissociation between structural integrity and functional adaptation. The principal findings of this study are as follows:

(1) Subclinical microvascular pathology: Hypobaric hypoxia induces progressive microscopic injury, predominantly peritubular capillary rarefaction and tubular damage, which occurs despite preserved gross organ morphology.

(2) Hemodynamic initiation: Glomerular morphometry exhibits a distinct biphasic response—acute reduction in glomerular diameter followed by compensatory hypertrophy—suggesting that the biphasic glomerular morphometric response is consistent with early hemodynamic adaptation and may contribute to downstream microvascular vulnerability.

(3) Exploratory biomarker pattern: CRE showed limited early discriminatory ability in the exploratory Day 3 ROC analysis, whereas KIM-1 showed the highest AUC among the predefined circulating biomarkers. However, AUC comparisons did not remain statistically significant after Tukey correction; therefore, the biomarker findings should be interpreted as hypothesis-generating rather than as validated diagnostic evidence.

(4) Structure–function uncoupling: Functional markers partially recovered or changed only later, whereas tubular injury and microvascular alterations persisted. We use the term compensated-fragility pattern only as a descriptive working term for this coexistence of apparently preserved or recovering serum functional markers with persistent structural injury, not as a formal disease entity or established pathological category.

(5) Critical biological inflection: Mathematical modeling identifies the acute phase (approximately the first week) as a critical non-linear inflection point, marking the transition from potentially reversible hemodynamic stress to established structural remodeling.

### 4.2 Macroscopic stability and subclinical pathology

A notable observation in our model was the dissociation between gross renal morphology and microscopic pathological alterations. Although absolute right kidney weight and kidney length did not differ significantly across groups, these parameters were not normalized to body weight and should therefore be interpreted with caution. The main finding of this section is that relatively stable gross anatomical indices can coexist with progressive microscopic renal injury during sustained hypobaric hypoxia.

This interpretation should be distinguished from observations in overt nephrotoxic, ischemia–reperfusion, or obstructive kidney injury models. These models differ substantially from hypobaric hypoxia in initiating mechanism, injury severity, inflammatory burden, and temporal course, and therefore should not be regarded as direct analogs of high-altitude renal injury. Nevertheless, they provide useful contextual contrast for interpreting gross renal measurements. For example, in ischemia–reperfusion injury, renal edema and volume expansion may accompany acute parenchymal damage, and ultrasound-derived kidney volume has been reported to reflect the severity and temporal evolution of tissue injury (Crislip et al., 2020). Conversely, clinical ultrasound studies also indicate that renal size alone has limited discriminatory value across renal disease states, emphasizing that macroscopic dimensions may not fully capture underlying parenchymal pathology (Ozmen et al., 2010).

In contrast to these direct renal injury models, hypobaric hypoxia represents a systemic and sustained exposure. Relevant chronic hypoxia studies have shown that low-oxygen exposure can induce subtle but progressive renal vascular and tubulointerstitial alterations, including arteriolar endothelial injury, vascular remodeling, tubulointerstitial inflammation, and tubular damage (Mazzali et al., 2003). Consistent with this concept, the present study found progressive tubular injury, reduced peritubular capillary density, and outer medullary erythrocyte accumulation/congestion-like changes despite stable kidney weight and length. Thus, the absence of significant macroscopic renal enlargement should not be interpreted as evidence of preserved renal structural integrity under hypobaric hypoxia.

Taken together, these findings support a cautious interpretation: gross renal morphology may remain relatively stable during sub-acute hypobaric hypoxia, whereas microscopic tubular and microvascular remodeling can still progress. This distinction is important because the present model is not equivalent to toxin-, ischemia–reperfusion-, or obstruction-induced kidney injury, but rather reflects a distinct form of systemic hypoxia-associated renal adaptation and injury.

### 4.3 Delayed serum creatinine response and early tubular injury markers

The limitations of traditional functional biomarkers were demonstrated by the inability of CRE to discriminate early histological injury at Day 3 (AUC = 0.49). The delayed CRE response most likely reflects the limited sensitivity of CRE during early renal injury and a subsequent reduction in filtration at later time points. In the present study, CRE increased progressively during prolonged hypoxic exposure, particularly at later time points, supporting its interpretation as a late functional signal rather than an early injury marker. Although hypobaric hypoxia may alter body weight, skeletal muscle mass, and creatinine generation, these variables were not measured and should therefore be considered potential confounders rather than proven mechanisms in this study (de Theije et al., 2018; Yin et al., 2024). The serum biomarker results should be interpreted with caution because of the small sample size and inter-animal variability. After Tukey correction, no individual pairwise comparison among the acute stress or functional serum biomarkers reached statistical significance. Therefore, the value of these markers in the present study lies primarily in their exploratory temporal patterns and their comparison with more persistent histological injury, rather than in definitive time-point-specific group differences.

In the exploratory Day 3 ROC analysis, KIM-1 showed the highest AUC among the predefined circulating biomarkers examined (AUC = 0.861, 95% CI: 0.641–1.000), whereas CRE showed no meaningful early discriminatory ability (AUC = 0.486, 95% CI: 0.124–0.848). However, the AUC confidence intervals were wide, and pairwise DeLong comparisons did not remain statistically significant after Tukey correction, including the comparison between KIM-1 and CRE (raw DeLong *P = 0.0204*; Tukey-adjusted *P = 0.0938*). Therefore, KIM-1 should be interpreted as a promising exploratory circulating marker in this experimental setting rather than as a validated or statistically superior diagnostic biomarker. KIM-1 is generally regarded as a marker of proximal tubular epithelial injury and has been investigated for the early detection of acute kidney injury (Seitkamal et al., 2025; Su et al., 2025). In the present dataset, KIM-1 showed the highest AUC for discriminating early histological injury at Day 3, whereas NGAL showed a lower AUC but may still provide complementary information on tubular stress. We therefore interpret KIM-1 as a promising exploratory circulating marker in this experimental setting, while recognizing that validation in larger cohorts, direct GFR-based studies, and urine-based assays is required before clinical translation.

IL-18 was included as an exploratory inflammatory marker to assess whether sustained hypobaric hypoxia was accompanied by a circulating inflammatory or inflammasome-related response. In contrast to KIM-1 and NGAL, serum IL-18 did not show a significant temporal change across exposure durations. This finding suggests that, under the present experimental conditions, early hypoxia-associated renal alterations were more clearly reflected by tubular stress markers than by a circulating IL-18 response. Although IL-18 showed a positive correlation with CysC, serum IL-18 may reflect systemic inflammatory or physiological stress rather than kidney-specific injury. Therefore, IL-18 should be interpreted as an exploratory systemic inflammatory readout rather than a primary marker of hypoxia-induced renal injury in this model.

### 4.4 Structure–function uncoupling: a compensated-fragility pattern

A central finding of this study is the temporal dissociation between serum functional markers and microscopic structural injury. In this manuscript, the term compensated-fragility pattern is used only as a descriptive working term to denote the coexistence of apparently preserved or partially recovering serum functional markers with persistent tubular injury and microvascular alterations. It is not intended to define a new disease entity, syndrome, or established pathological category.

Although Tukey-adjusted pairwise comparisons did not confirm a significant Day 7 increase, CysC showed a numerical and modeled non-linear trajectory, with a rise around Day 7 followed by subsequent decline. In contrast, structural injury indices, including tubular injury and the CD34-defined reduction in the PTC/tubule ratio, remained evident during prolonged hypoxic exposure. CRE changed later and was relatively insensitive during the earliest phase of exposure. This pattern suggests that conventional serum functional markers may underestimate ongoing microscopic renal injury during sub-acute hypobaric hypoxia.

Morphometric data provide a structural context for this dissociation. Glomerular diameter exhibited a biphasic response, with an early reduction at Day 3 followed by subsequent enlargement at later time points. The early reduction may reflect reduced intraglomerular perfusion, partial capillary tuft collapse, or altered glomerular filling, whereas the later enlargement may be compatible with adaptive glomerular remodeling. However, because direct renal blood flow, intraglomerular pressure, single-nephron GFR, and renal tissue oxygenation were not measured, these morphometric changes should not be interpreted as direct evidence of a specific hemodynamic mechanism.

The later increase in glomerular diameter may be viewed in the context of adaptive filtration responses described in other kidney disease settings. Classical and contemporary studies of glomerular hyperfiltration indicate that increased single-nephron filtration can help maintain overall renal clearance when functional nephron reserve is reduced, but sustained hyperfiltration may also impose long-term structural and metabolic burdens (Hostetter et al., 1981; Cortinovis et al., 2022; Kanbay et al., 2024). Increased filtration and tubular transport workload may further increase renal oxygen demand, particularly in metabolically active nephron segments (Vallon and Thomson, 2020). In the present model, this framework provides a physiological context rather than direct proof of hyperfiltration. Thus, animals with apparently preserved or partially recovering serum CysC or CRE levels may still retain persistent microscopic tubular and microvascular abnormalities, suggesting reduced renal reserve under sustained hypobaric hypoxia.

Within this limited framework, the compensated-fragility pattern may be useful as a hypothesis-generating description of structure–function uncoupling during sub-acute hypobaric hypoxia. This interpretation requires validation in studies incorporating direct GFR measurement, urinary biomarkers, renal blood-flow and oxygenation assessment, perfusion-controlled histology, additional endothelial markers, and recovery experiments.

### 4.5 Microvascular rarefaction and medullary congestion

Correlation analysis mapped a hierarchy of injury in which microvascular integrity links early tissue stress, medullary hemodynamics, and functional decline (Lin et al., 2025; Ren et al., 2025; Chen et al., 2025). The inverse correlation between PTC density and KIM-1, CRE, and tubular injury scores indicates that microvascular loss is associated with early renal deterioration.

It should be emphasized that peritubular capillary rarefaction has not been consistently defined in the literature as a typical or diagnostic histopathological feature of chronic hypobaric hypoxia. Studies of high-altitude renal physiology and high-altitude renal syndrome have more commonly focused on altered glomerular filtration, erythrocytosis-related hemodynamic stress, hyperuricemia, proteinuria, glomerular hypertrophy, podocyte injury, glomerulosclerosis, and arterial or arteriolar remodeling rather than on quantitative loss of the peritubular capillary network. In contrast, peritubular capillary rarefaction is a well-recognized feature in chronic progressive kidney disease and in several experimental models of AKI-to-CKD transition or renal fibrosis. Therefore, the CD34-defined reduction in the PTC/tubule ratio observed in the present 3–28-day hypobaric hypoxia model should be interpreted as a model-specific and hypothesis-generating finding, rather than as an established hallmark of chronic high-altitude renal disease. This distinction is important because our study examined a sub-acute exposure window under controlled experimental conditions, whereas chronic high-altitude kidney disease in humans is shaped by long-term erythrocytosis, hyperviscosity, systemic hemodynamic adaptation, comorbidities, and genetic or environmental modifiers.

The early reduction in glomerular diameter observed at Day 3 provides a morphometric indication of altered glomerular architecture during the initial phase of hypoxic exposure. In two-dimensional histological sections, a smaller glomerular profile may reflect reduced intraglomerular capillary filling, altered glomerular perfusion pressure, or partial capillary tuft collapse. However, this measurement cannot distinguish afferent from efferent arteriolar tone, nor can it establish segment-specific changes in renal vascular resistance. Therefore, we interpret the early glomerular morphometric change as a structural correlate of altered glomerular perfusion rather than as direct evidence of afferent arteriolar vasoconstriction.

Because the renal microvasculature is arranged in series, changes in glomerular perfusion may influence downstream post-glomerular and peritubular capillary blood supply. In the present study, the temporal coexistence of early glomerular morphometric alteration and reduced CD34-defined PTC/tubule ratio suggests a possible association between altered glomerular perfusion and downstream microvascular remodeling. However, causality and arteriolar segment-specific mechanisms cannot be determined without direct measurements of renal blood flow, intraglomerular pressure, afferent and efferent arteriolar resistance, or renal tissue oxygenation.

Furthermore, the inverse association between PTC density and outer medullary congestion (ρ = -0.88) suggests that capillary rarefaction contributes to medullary circulatory stasis (Ren et al., 2023). Loss of the peritubular microvasculature reduces both oxygen delivery and metabolic clearance in the tubulointerstitial compartment (Nangaku, 2006; Zhang X. et al., 2025), thereby predisposing the outer medulla, an intrinsically hypoxia-vulnerable region, (Chen et al., 2009; Heyman et al., 2020)-to circulatory stasis. Once established, medullary congestion may further exacerbate local hypoxia by increasing intravascular resistance and tissue pressure, amplifying tubular injury (McLarnon et al., 2023) and reinforcing microvascular dysfunction. By reducing both oxygen delivery and erythrocyte clearance, PTC loss predisposes the hypoxia-sensitive outer medulla to congestive injury, which further amplifies tubular damage. Within this framework, outer medullary congestion acts as a secondary hemodynamic response, while functional decline manifests as the downstream consequence of these integrated processes.

**Figure 8:**
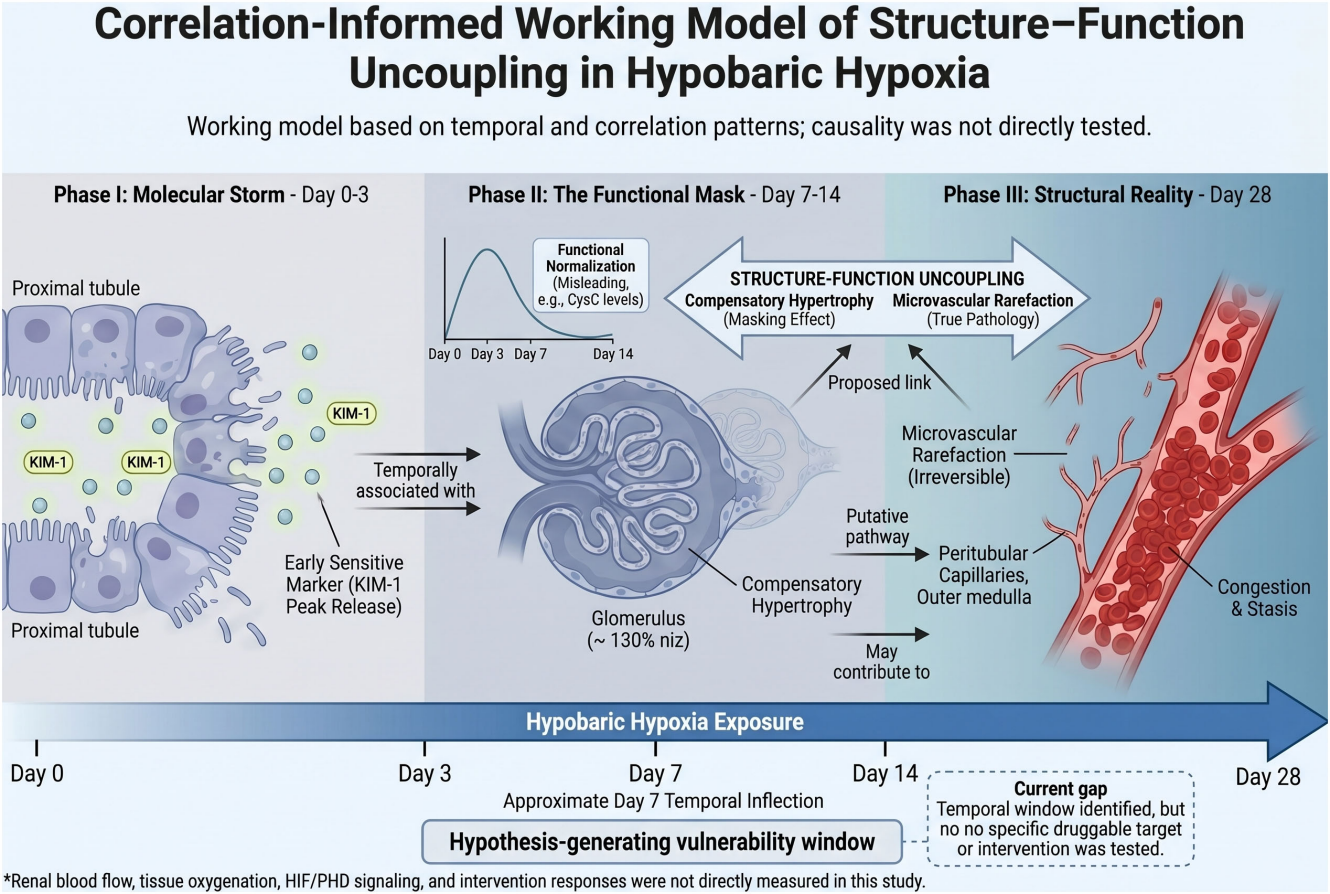
Correlation-informed working model of structure–function uncoupling during sub-acute hypobaric hypoxia. This schematic summarizes the temporal and correlation patterns observed in this study, including early glomerular morphometric change, peritubular capillary rarefaction, tubular injury, medullary congestion, and delayed functional responses. The model is intended as a hypothesis-generating framework and does not establish a definitive causal pathway. The approximate Day 7 inflection represents a potential early vulnerability window rather than a validated therapeutic threshold. Although this temporal pattern suggests that renal microvascular preservation may be a plausible direction for future studies, the present study did not identify a specific druggable molecular target or test an intervention.

Collectively, these findings support a working model in which early glomerular morphometric changes, altered glomerular perfusion, reduced CD34-defined PTC/tubule ratio, and tubular injury are temporally linked during sub-acute hypobaric hypoxia. In this framework, early changes in glomerular architecture may coincide with downstream microvascular alterations, which may in turn contribute to outer medullary erythrocyte accumulation/congestion-like changes and tubular injury. However, this model remains hypothesis-generating. The present data do not establish whether afferent or efferent arteriolar tone predominates, nor do they prove that altered arteriolar resistance is the initiating mechanism. These findings are summarized in our working model (**Figure 8**).

### 4.6 Temporal dynamics and non-linear inflection identified by RCS analysis

RCS modeling revealed that hypoxia-induced renal injury does not progress at a constant rate, but rather evolves through temporally staged phases. A consistent non-linear inflection point emerged at approximately Day 7 for multiple parameters, including CysC, glomerular diameter, and histopathological injury scores. Prior to this point, the estimated slopes were steeply positive for several structural and functional readouts, suggesting an early phase of dynamic renal adaptation and injury-related change. Beyond Day 7, the slopes attenuated significantly, signaling a transition toward slower progression and maladaptive tissue remodeling. The convergence of these inflection points highlights the first week of exposure as a biologically constrained period of heightened susceptibility, during which renal injury progresses most rapidly.

Furthermore, the RCS analysis uncovered a critical kinetic divergence among functional biomarkers. While CysC exhibited a highly significant non-linear relationship (P-nonlinear = 0.003), CRE demonstrated a predominantly linear association with exposure duration (P-nonlinear = 0.229). This divergence reflects inherent differences in physiological sensitivity. CRE changes gradually (Ronco et al., 2019) and is known to have limited sensitivity during early renal injury; in the present study, its later increase is more consistent with delayed functional involvement rather than early injury detection. In contrast, CysC may capture subtle early changes in filtration more readily than CRE and is less dependent on muscle mass (Haines et al., 2023; Xu et al., 2023); CysC may be more responsive than CRE to early changes in filtration, but the present findings should be interpreted cautiously because Tukey-adjusted pairwise comparisons did not confirm significant time-point-specific differences.

Collectively, the RCS data provide a quantitative framework, demonstrating that renal adaptation to high-altitude hypoxia is not a linear continuum, but a staged process driven by distinct early acute and later chronic mechanisms. Furthermore, these contrasting kinetic profiles underscore the necessity of integrating multiple functional indices, rather than relying on a single biomarker, to accurately capture the complex temporal dynamics of hypoxia-induced renal injury. The Day 7 inflection point should be interpreted as a hypothesis-generating temporal window rather than a definitive therapeutic threshold. The current data suggest that the first week of hypoxic exposure may represent a period of heightened biological activity, during which interventions aimed at preserving endothelial viability, stabilizing microvascular perfusion, modulating hypoxia-response pathways, or limiting inflammatory amplification may be biologically plausible. However, this study did not measure specific molecular targets or test any intervention. Therefore, the targetable mechanisms underlying this temporal window remain undefined.

### 4.7 Translational implications

These findings offer specific translational considerations for high-altitude medicine, particularly regarding early screening and risk stratification.

First, the temporal screening window must be carefully calibrated to account for interspecies metabolic scaling. While our RCS analysis identified Day 7 as a critical biological inflection point in rats, the accelerated metabolic rate and cellular turnover of rodents imply that this window represents an acute-to-subacute transition state, rather than a literal seven-day calendar target for humans. Clinically, this “inflection window” likely corresponds to the initial days-to-weeks of human high-altitude acclimatization. Consequently, reliance on functional measurements taken after several weeks—when adaptive functional normalization may have already occurred—could miss the critical period of microvascular regression. Screening should therefore be intensified during the earliest phase of ascent.

Second, risk stratification for high-risk plateau populations, such as soldiers and railway workers, should not rely on CRE alone. In the present model, CRE showed limited early discriminatory ability, whereas KIM-1 showed the highest AUC among the predefined circulating biomarkers in the exploratory Day 3 ROC analysis. However, AUC confidence intervals were wide, and DeLong pairwise comparisons did not remain statistically significant after Tukey correction. Therefore, these findings should be interpreted as hypothesis-generating rather than as evidence of validated diagnostic performance.

These results suggest that tubular stress markers may be worth further investigation as adjunctive indicators of early hypoxia-associated renal vulnerability, but they cannot yet be recommended as a validated screening panel. Further validation in larger animal studies, urine-based assays, direct GFR-based assessments, and human high-altitude cohorts is required before clinical application. In this context, the term “structural biomarkers” does not imply routine kidney biopsy for clinical screening. Rather, it refers to measurable indicators that may reflect tubular or microvascular injury, including circulating or urinary tubular stress markers such as KIM-1 and NGAL, and potentially non-invasive imaging indices in future studies.

Third, the observed structure–function uncoupling may help inform future preventive studies during high-altitude exposure. In the present model, the CD34-defined reduction in the PTC/tubule ratio was associated with tubular injury and outer medullary erythrocyte accumulation/congestion-like changes, suggesting that microvascular alterations may be linked to persistent microscopic renal injury during sub-acute hypobaric hypoxia. However, because PTC rarefaction is not yet established as a typical histopathological feature of chronic hypobaric hypoxia, this finding should be interpreted as model-specific and hypothesis-generating. Future studies should examine whether strategies aimed at preserving renal microvascular integrity during early hypoxic exposure can attenuate subsequent structural injury. Importantly, the present study did not test any intervention or identify a specific druggable target; therefore, this preventive implication remains speculative. Rather than implying a kidney-specific intervention alone, these findings support the need to consider renal protection as one component of broader multi-organ monitoring and protection during high-altitude exposure.

### 4.8 Limitations and future directions

Several limitations warrant consideration. First, the major limitation of this study is the absence of time-matched normoxic control groups for each hypoxia exposure duration. A single normoxic control group was used as the reference group. Therefore, time-related factors unrelated to hypoxia, including age, handling, chamber-related procedures, and potential assay drift, cannot be fully excluded. The temporal trends observed in this study should therefore be interpreted as hypoxia-associated patterns rather than definitive effects proven against time-matched normoxic controls. This study did not include a normoxic recovery phase after hypobaric hypoxia exposure. Therefore, we cannot determine whether the observed microvascular and tubular alterations are reversible after return to normoxia or whether they progress to persistent structural injury. Future recovery experiments are needed to distinguish adaptive remodeling from lasting damage.

Second, Renal functional assessment was incomplete. Although serum CRE and CysC were measured as functional surrogates, direct GFR measurement using inulin clearance or comparable approaches was not performed. In addition, urine was not collected, which precluded assessment of urinary albumin, albumin-to-creatinine ratio, urinary KIM-1, urinary NGAL, or other urinary injury markers. Therefore, the proposed structure-function dissociation should be interpreted as suggestive rather than definitive. The RTI score was used as a semi-quantitative measure of overall hypoxia-associated tubular injury and was not designed to distinguish strictly between acute and chronic tubular lesions. Although the same criteria were applied consistently and blindly across groups, future studies using additional markers of apoptosis, proliferation, fibrosis, and tubular atrophy are needed to better characterize the temporal nature of tubular injury. Future studies should include Sirius Red or collagen staining and F4/80 immunohistochemistry to define whether the observed microvascular changes are accompanied by fibrosis and inflammatory cell infiltration. Another important limitation is that PTC rarefaction was assessed using CD34 immunohistochemistry alone. Although CD34 is widely used as an endothelial marker, reduced CD34-positive profiles may reflect true capillary loss, altered endothelial antigen expression, section-plane effects, or tissue-processing-related variation. In addition, renal tissues were not evaluated using additional endothelial markers such as CD31 or endomucin, nor was microvascular perfusion assessed by perfusion-based labeling techniques. Therefore, the observed reduction in the PTC/tubule ratio should be regarded as preliminary evidence of microvascular alteration in this model. Future studies should combine multiple endothelial markers, perfusion-controlled fixation or vascular labeling, direct renal oxygenation assessment, and longer exposure or recovery time courses to determine whether PTC rarefaction is reproducible and whether it generalizes to chronic hypobaric hypoxia.

Third, body weight was not consistently recorded across all experimental groups, which precluded normalization of kidney weight to body weight. Given that hypobaric hypoxia may induce systemic metabolic alterations, including potential weight loss and muscle catabolism, this limitation may contribute to variability in macroscopic parameters and influence the interpretation of creatinine-based functional indices. However, the primary conclusions of this study are derived from histopathological and microvascular parameters (e.g., PTC/tubule ratio, RTI score), which are independent of body weight normalization. Accordingly, macroscopic parameters should be interpreted as supportive rather than central to the mechanistic conclusions. In addition, only the right kidney was analyzed in this study to maintain sampling consistency, which may not fully capture potential intra-animal variability between kidneys.

Fourth, because hypobaric hypoxia is a systemic exposure, multi-organ stress may have contributed to the observed biomarker and pathological changes. We did not assess terminal heart weight, heart weight-to-tibia length ratio, lung histology, or pulmonary injury markers. Therefore, the present findings should be interpreted within a kidney-focused framework, and future studies should include cardiopulmonary assessments to determine whether renal injury occurs independently or as part of a broader multi-organ response.

Fifth, while medullary congestion implies altered renal venous hemodynamics, right atrial and renal venous pressures were not measured. In addition, renal tissues were immersion-fixed rather than perfusion-fixed. Therefore, erythrocyte accumulation within outer medullary vessels may have been influenced by terminal blood collection, tissue handling, or post-excision vascular retention. Accordingly, the outer medullary congestion score should be interpreted as a semi-quantitative morphological observation rather than definitive evidence of *in vivo* vasa recta congestion or renal venous outflow impairment.

Sixth, no formal *a priori* sample size calculation was performed. Therefore, the study should be interpreted as an exploratory animal study, and the findings require validation in larger, adequately powered cohorts. In addition, serum biomarker analyses were limited by the small number of animals per group and by substantial inter-animal heterogeneity. After Tukey correction, individual pairwise comparisons among the acute stress and functional serum biomarkers did not reach statistical significance. Therefore, these biomarker findings should be interpreted as exploratory temporal patterns rather than definitive time-point-specific differences. Larger, prospectively powered studies are required to validate the temporal behavior and discriminatory performance of these circulating markers.

Finally, the specific molecular crosstalk between the tubular epithelium and the endothelium remains to be defined. Future studies incorporating single-cell transcriptomics and targeted vascular interventions during the critical Day 3–7 window will be essential to validate the “Compensated Fragility” framework. We did not directly measure renal blood flow, renal tissue oxygenation, HIF-family proteins, prolyl hydroxylase activity, or other oxygen-sensing pathways. In addition, glomerular diameter was measured on two-dimensional histological sections and should be interpreted as a morphometric index rather than a direct hemodynamic measurement. Smaller glomerular profiles may reflect reduced intraglomerular capillary filling, altered perfusion pressure, section-plane effects, or partial tuft collapse. Because renal blood flow, intraglomerular pressure, afferent and efferent arteriolar tone, and renal oxygenation were not directly measured, the present study cannot determine the arteriolar segment responsible for the observed glomerular morphometric changes. Therefore, the proposed hemodynamic and hypoxia-response mechanisms should be considered hypotheses for future investigation rather than mechanisms directly demonstrated by the present data.

## 5 Conclusion

This study describes the temporal dynamics of renal structural and functional alterations during sustained hypobaric hypoxia in rats. The findings suggest that relatively preserved gross renal morphology and delayed changes in conventional functional markers may coexist with progressive microscopic injury, including tubular injury, outer medullary erythrocyte accumulation/congestion-like changes, and a CD34-defined reduction in the peritubular capillary (PTC)/tubule ratio in this sub-acute rat model. Because PTC rarefaction has not yet been established as a typical histopathological feature of chronic hypobaric hypoxia, this microvascular observation should be interpreted as model-specific and hypothesis-generating rather than as a canonical lesion of high-altitude renal disease.

KIM-1 showed the highest exploratory Day 3 AUC among the circulating biomarkers examined, whereas CRE showed limited early discriminatory ability. However, the AUC estimates had wide confidence intervals and pairwise DeLong comparisons did not remain statistically significant after Tukey correction. Therefore, these circulating biomarker findings should be interpreted as hypothesis-generating rather than as validated diagnostic evidence. The observed biphasic glomerular morphometric response and progressive microvascular alterations support a working hypothesis of structure–function uncoupling during sub-acute hypobaric hypoxia, although direct hemodynamic, oxygenation-related, and molecular mechanisms remain to be defined. RCS analysis identified an approximate Day 7 temporal inflection, which should be interpreted as a hypothesis-generating temporal pattern rather than a definitive biological or therapeutic threshold.

Overall, these findings support the potential value of combining conventional functional markers with tubular stress markers and microvascular injury-related indicators to detect early renal vulnerability during hypobaric hypoxia. Further studies incorporating time-matched normoxic controls, direct GFR assessment, urinary biomarkers, systemic physiological readouts, cardiopulmonary evaluation, additional endothelial markers, perfusion-controlled histology, direct renal oxygenation measurements, and longer exposure or recovery protocols are needed to validate these observations and determine whether the observed microvascular alteration represents a reproducible component of high-altitude renal remodeling.

## Data Availability

The raw data supporting the conclusions of this article will be made available by the authors, without undue reservation.
